# Clinical Outcome and Safety of Combined Radiation Therapy and Maintenance Avelumab for Bladder Cancer: A Case Series

**DOI:** 10.7759/cureus.94938

**Published:** 2025-10-19

**Authors:** Daiki Ikarashi, Yasushi Nozaki, Koyo Kikuchi, Hisanori Ariga, Wataru Obara

**Affiliations:** 1 Department of Urology, Iwate Medical University Hospital, Morioka, JPN; 2 Department of Radiation Oncology, Iwate Medical University Hospital, Morioka, JPN

**Keywords:** avelumab, bladder cancer, combination, maintenance therapy, radiation

## Abstract

The combination of maintenance avelumab and radiation therapy (RT) has emerged as a promising approach for enhancing therapeutic efficacy. However, clinical evidence on the use of this combination therapy is limited. This single-institution case series includes five patients with locally advanced or metastatic urothelial carcinoma who received maintenance avelumab combined with RT for primary bladder cancer. The characteristics of the patients, number of avelumab cycles received upon RT initiation, RT dose/delivery approach, severity and type of avelumab or radiation treatment-related adverse events, and response to RT were recorded. The primary site in all patients was the bladder. Among the patients receiving avelumab maintenance therapy, four underwent RT to the primary lesion for local control and one for bladder preservation. The patients only developed grade 1 RT-related adverse events without treatment interruptions. All patients had a complete response at the irradiation site of the primary bladder cancer. One patient died of community-acquired pneumonia, and the others are still being treated. The combination of maintenance avelumab and RT to the primary site could be effective and safe for local control in patients with bladder cancer.

## Introduction

Urothelial cancer (UC) is one of the most common malignancies, with a high rate of recurrence and a substantial need for better treatment strategies [1]. Despite advancements in surgical techniques and chemotherapy, the prognosis of patients with advanced-stage or metastatic bladder cancer remains poor, with a five-year survival rate of <15% in those with distant metastases [2]. In recent years, immune checkpoint inhibitors (ICIs) targeting the programmed cell death protein 1 (PD-1)/programmed death-ligand 1 (PD-L1) axis have emerged as a novel therapeutic strategy for advanced-stage UC, showing promising clinical activity and manageable toxicity profiles.

The potential efficacy of avelumab, a fully human IgG1 monoclonal antibody targeting PD-L1, for treating UC has been investigated [3]. The JAVELIN Bladder 100 trial, a randomized, phase III study, evaluated the efficacy of maintenance avelumab therapy after first-line platinum-based chemotherapy in patients with locally advanced or metastatic UC (la/mUC) [4]. In this pivotal trial, maintenance avelumab was associated with a significantly better overall survival (OS) compared with best supportive care alone. The median OS rates were 21.4 months in the avelumab group and 14.3 months in the control group (hazard ratio (HR): 0.69, 95% confidence interval (CI): 0.56-0.86; p = 0.001). Based on this finding, avelumab can be the novel standard of care in this setting [4,5]. However, the confirmed objective response rate was 9.7%. Thus, avelumab alone had a limited efficacy. Therefore, the efficacy of new treatment strategies using avelumab in achieving long-term response should be explored.

Radiation therapy (RT) exerts its antitumor effects primarily by inducing DNA damage in cancer cells, leading to cell death. Additionally, RT can modulate the tumor microenvironment, enhancing antigen presentation and promoting immune-mediated tumor control, which may synergize with immunotherapy [6]. Considering the immunomodulatory effects of RT, the use of RT along with PD-L1 inhibitors has gained increasing attention. However, the efficacy of maintenance avelumab plus RT for la/mUC remains unclear. This case series aimed to examine the potential of avelumab as a maintenance therapy in combination with RT for la/mUC.

## Case presentation

Methods

This protocol for this case series was approved by the Institutional Review Board of Iwate Medical University Hospital, Japan (approval no. MH2023-059). The study was performed in accordance with the Declaration of Helsinki, and all patients provided written informed consent.

Five patients who received maintenance avelumab and RT for advanced-stage or metastatic bladder cancer at Iwate Medical University Hospital between November 2023 and April 2025 were examined. All patients had histologically or cytologically confirmed UC. Patients who received concomitant RT for local control of the primary tumor with curative intent during avelumab maintenance therapy were included in this analysis. Cases that received palliative irradiation were excluded. Patients received maintenance avelumab after at least four cycles of combined gemcitabine and cisplatin or combined gemcitabine and carboplatin as 1L chemotherapy. Then, they were radiologically confirmed as having stable disease (SD) or better. During the study period, avelumab 10 mg/kg was routinely administered every two weeks until disease progression or discontinuation because of major adverse events.

The radiation delivery approach was three-dimensional conformal RT for all patients. In accordance with the institutional protocols, the anatomical site and radiation dose to the primary bladder were determined by two board-certified radiation oncologists (KK and HA), taking into account both clinical indications and patient-specific anatomical factors. The concurrent administration of avelumab and RT on the same day was permitted.

The characteristics of the patients, number of avelumab cycles received during RT initiation, location of RT sites, RT dose, severity and type of treatment-related adverse events (TRAEs) including RT and avelumab, response to RT, and date of the most recent visit to our institution were recorded. TRAEs were retrospectively collected from patients’ medical records, including clinical notes and laboratory data.

According to the Response Evaluation Criteria in Solid Tumors (RECIST) 1.1 criteria, disease response to treatment was defined as complete response, partial response, SD, and progressive disease [7]. Follow-up was defined as the interval from RT completion to the date of the most recent visit to our hospital. OS was defined as the period from avelumab initiation to the date of death or last follow-up visit.

Results

Table 1 shows the characteristics of the patients. The median age at avelumab initiation was 68 (range: 59-78) years, and all patients were male. The median follow-up period was 11.2 (range: 6-27) months. All patients received bladder RT. Only one patient had additional RT for lymph node metastases. This patient was initially diagnosed with cT4N0M0 disease and subsequently received maintenance therapy with avelumab after chemotherapy. However, due to the progression of the primary lesion and the development of metastasis in the obturator lymph node, RT was administered to each site.

**Table 1:** Patients characterisics Cases 1–3 and 5 represent patients in whom metastatic lesions were controlled, and radiotherapy was administered to the primary tumor. Case 4 represents a patient in whom radical cystectomy was attempted following neoadjuvant chemotherapy, but bladder sparing was ultimately achieved. ECOG PS, Eastern Cooperative Oncology Group Performance Status; GC, gemcitabine + cisplatin; SD, stable disease; PR, partial response; CR, complete response; RT, radiation therapy; Gy, Gray; Fr, fraction; 3D-CRT, 3-dimensional conformal radiation therapy; IMRT, Intensity modulated radiation therapy; OS, overall survival

Case	Age	ECOG PS	Primary site	cT stage	Chemotherapy regimen	Cycle	Chemo- efficacy	Avelumab cycles at RT	RT dose	RT Fr	RT sites	RT delivery	RT efficacy	Outcome	OS (months)
1	78	0	bladder	cT4N0M0	GC	4	SD	14	60Gy	30	bladder Rt. Obturator LN	3D-CRT	CR	Death	18
2	68	1	bladder	cT3N0M1	GC	4	PR	54	55Gy	20	bladder	3D-CRT	CR	Alive	40
3	72	0	bladder	cT4N3M0	GC	4	SD	16	55Gy	20	bladder	3D-CRT	CR	Alive	35
4	63	0	bladder	cT2N0M0	GC	6	SD	1	55Gy	20	bladder	IMRT	CR	Alive	25
5	59	1	bladder	cT3N3M1a	GC	4	SD	12	55Gy	20	bladder	3D-CRT	CR	Alive	25

**Figure 1:**
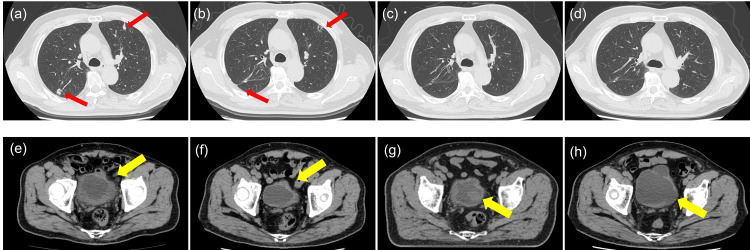
Representative case managed with avelumab and radiation (Case 2) Computed tomography (CT) scan revealed shrinkage and increase of the primary bladder cancer (yellow arrow) and lung metastasis (red arrow) in case 2 (a, e). Images at pretreatment (b, f), after four cycles of GC (c, g), and after 54 cycles of avelumab therapy (d, h). After additional radiation combined with avelumab therapy, there was maintenance of tumor shrinkage for the primary bladder cancer and lung metastasis for 27 months.

The most common indications for RT were local primary tumor control in four patients and bladder sparing in one who refused cystectomy during neoadjuvant chemotherapy. All patients completed three-dimensional conformal RT. Four of these patients, patients with locally advanced or metastatic bladder cancer, received four cycles of first-line gemcitabine and cisplatin (GC) chemotherapy. Given that SD or better was achieved by GC, maintenance therapy with avelumab was subsequently initiated. Metastatic lesions of these cases were under control during treatment with avelumab. However, only the primary lesion got enlarged (Figure 1). Therefore, RT to the primary bladder cancer was performed for local control.

**Figure 2:**
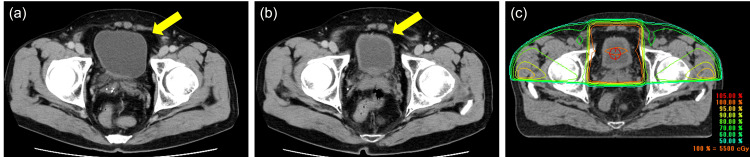
Case managed by bladder sparing therapy with avelumab and radiotherapy (Case 4) Computed tomography (CT) scan showed the primary bladder tumor (yellow arrow). (a) Pre-NAC after TURBT, (b) post-NAC, and (c) image of 3D-CRT along with isodose lines. 3D-CRT, three-dimensional conformal radiation therapy. TURBT, transurethral resection of bladder tumor; NAC, neoadjuvant chemotherapy; 3D-CRT, three-dimensional conformal radiation therapy

The one remaining patient received RT for primary bladder cancer with maintenance avelumab as bladder sparing therapy. The rationale for combining maintenance avelumab therapy with RT was that the effect of neoadjuvant chemotherapy on the primary lesion was considered insufficient. The patient’s response to the six cycles of previous chemotherapy was assessed as SD. However, a slight increase in the size of the primary lesion was observed. Therefore, after a multidisciplinary discussion with the radiation oncology team, a treatment strategy combining RT as a bladder-preserving approach with subsequent avelumab maintenance therapy was adopted (Figure 2).

**Figure 3:**
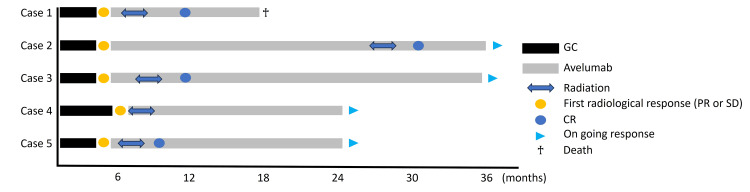
Visual timelines of all cases. Except for one patient who died, all remaining cases demonstrated sustained treatment efficacy. GC, gemcitabine + cisplatin; PR, partial response; SD, stable disease; CR, complete response

The median OS was 25 months (range: 18-40), and all patients, except one who died of community-acquired pneumonia, are still receiving avelumab for controlling the primary and metastatic lesions. A patient who underwent bladder sparing was followed up for 25 months without recurrence or metastasis. In all cases, the antitumor effect of RT for primary bladder cancer was a complete response (Figure 3).

Table 2 shows the TRAEs, including immune-related adverse events induced by avelumab and RT-related adverse events, respectively. All TRAEs were grade 1, and there were no treatment interruptions attributed to TRAEs. Only two patients developed radiation-related adverse events, both related to urination during irradiation.

**Table 2:** Treatment-related adverse events Adverse events are categorized as immune-related or radiation-related. Grades are reported according to the Common Terminology Criteria for Adverse Events (CTCAE) version 5.0. irAE, immune-related adverse event; AE, adverse event; RT, radiotherapy

Case	irAEs (Grade)	AEs related to RT (Grade)
1	Rash (G1)	-
2	Infusion reaction (G1), Rash (G1)	Urinary frequency (G1)
3	-	-
4	Diarrhea (G1)	-
5	-	Dysuria (G1)

## Discussion

To the best of our knowledge, this is the first case series exploring the combined use of RT to the primary bladder cancer and maintenance avelumab for la/mUC. Notably, no unexpected or severe complications were observed during RT administered concurrently with maintenance avelumab. Meanwhile, a high antitumor effect was achieved in this case series.

The combination of ICIs and RT is a promising therapeutic strategy for managing UC. Previous studies have revealed that RT may have a synergistic effect when used alongside ICIs, potentially enhancing systemic antitumor immunity [8,9]. In the context of UC, where immune checkpoint blockade has shown durable responses in a subset of patients with metastatic disease, the addition of localized RT may further increase therapeutic efficacy, particularly by targeting residual primary lesions. Fukushima et al. showed that 17 patients who were previously exposed to RT to the primary tumor during pembrolizumab had superior OS and objective response rate compared with 81 patients who did not receive RT. They also revealed that RT to the primary tumor might enhance the efficacy of pembrolizumab [10].

The application of RT to the primary tumor site after systemic disease control achieved via ICIs is an area of particular interest. If ICIs can effectively control metastatic lesions, administering RT to the primary tumor may contribute not only to local control but also to the amplification of systemic immune responses via the so-called abscopal effect [11]. This phenomenon, in which localized radiation induces regression of distant, non-irradiated tumors, is believed to be mediated by enhanced antigen presentation and T-cell activation, which are processes already potentiated by ICIs. Thus, RT may be a means of reinforcing and sustaining the antitumor immune milieu established by prior checkpoint inhibition [12]. Further, delivering RT in a setting of minimal residual disease, after systemic disease burden has been reduced by ICIs, may optimize the immunomodulatory effects of radiation. Under these conditions, the immune system, which is unburdened by extensive tumor load, may be more responsive to radiation-induced signals, thereby increasing the likelihood of effective immunological memory and durable control. Future prospective studies should be conducted to evaluate the optimal sequencing and timing of RT in relation to ICI administration, and to identify the predictive biomarkers of response to combination therapy.

A notable advantage of avelumab over other ICIs lies in its favorable toxicity profile. Unlike anti-CTLA-4 and anti-PD-1 antibodies such as ipilimumab and pembrolizumab, avelumab is associated with lower incidence rates of immune-related adverse events, possibly due to its IgG1 isotype and shorter half-life [13]. Due to this profile, it can be particularly suitable when combined with RT, which itself may induce inflammatory responses. Moreover, avelumab exerts antitumor effects not only through ICIs but also via antibody-dependent cellular cytotoxicity (ADCC), a mechanism mediated primarily by natural killer cells [14]. Unlike other anti-PD-1/PD-L1 antibodies that are engineered to decrease Fc receptor binding, avelumab retains an intact Fc region, thereby enabling ADCC activity and providing an additional immune-mediated antitumor mechanism [15]. Considering this background, the combination of RT and avelumab would be beneficial. RT may enhance the susceptibility of tumor cells to ICIs and simultaneously enhance the recruitment and activation of effector immune cells, including NK cells, thereby potentiating ADCC mediated by avelumab. Further, preclinical evidence has shown that RT can modulate the tumor stroma and vasculature to facilitate immune cell infiltration, potentially synergizing with avelumab to achieve improved local and systemic tumor control [16].

Taken together, although direct evidence is still emerging, the combination of avelumab maintenance therapy and RT may represent a rational therapeutic strategy for patients with UC, particularly those requiring local control. Prospective clinical trials should be performed to evaluate the efficacy and safety of this approach and to identify biomarkers that predict response.

A limitation of this case series was that conclusions cannot be drawn because of the limited sample size, retrospective design, short follow-up periods, and lack of a control group. In addition, biopsies were not performed before and after RT. Therefore, we were unable to evaluate the changes in immune response in the tumor immune microenvironment due to RT. Thus, a future prospective study with a large cohort and evaluation of biological correlations would validate the results of this exploratory study. However, we believe that the clinical information presented in this case series can contribute to further understanding the potential therapeutic spectrum of combined RT and maintenance avelumab for la/mUC.

## Conclusions

The combination of avelumab, PD-L1 inhibitors, and RT is a promising maintenance treatment strategy for bladder cancer. By leveraging the immunostimulatory effects of radiation along with immune checkpoint blockade, this approach can enhance antitumor immunity and improve patient outcomes. Further clinical studies should be conducted to refine treatment protocols and establish this combination as a standard therapeutic option for bladder cancer.
